# Molecular surveillance of mutations in the cytochrome b gene of *Plasmodium falciparum *in Gabon and Ethiopia

**DOI:** 10.1186/1475-2875-5-112

**Published:** 2006-11-21

**Authors:** Tamirat Gebru, Asrat Hailu, Peter G Kremsner, Jürgen FJ Kun, Martin P Grobusch

**Affiliations:** 1 Department of Medical Laboratory Technology, Faculty of Health Sciences, Haramaya University, Haramaya, Ethiopia; 2 Medical Research Unit, Albert Schweitzer Hospital, Lambaréné, Gabon; 3 Department of Microbiology, Immunology and Parasitology, Faculty of Medicine, Addis Ababa University, Addis Ababa, Ethiopia; 4 Department of Parasitology, Institute of Tropical Medicine, University of Tübingen, Tübingen, Germany; 5 Infectious Diseases Unit, Division of Clinical Microbiology and Infectious Diseases, National Health Laboratory Service and School of Pathology, Faculty of Health Sciences, University of the Witwatersrand, Johannesburg, South Africa

## Abstract

**Background:**

Atovaquone is part of the antimalarial drug combination atovaquone-proguanil (Malarone^®^) and inhibits the cytochrome bc_1 _complex of the electron transport chain in *Plasmodium *spp. Molecular modelling showed that amino acid mutations are clustered around a putative atovaquone-binding site resulting in a reduced binding affinity of atovaquone for plasmodial *cytochrome b*, thus resulting in drug resistance.

**Methods:**

The prevalence of *cytochrome b *point mutations possibly conferring atovaquone resistance in *Plasmodium falciparum *isolates in atovaquone treatment-naïve patient cohorts from Lambaréné, Gabon and from South Western Ethiopia was assessed.

**Results:**

Four/40 (10%) mutant types (four different single polymorphisms, one leading to an amino acid change from M to I in a single case) in Gabonese isolates, but all 141/141 isolates were wild type in Ethiopia were found.

**Conclusion:**

In the absence of drug pressure, spontaneous and possibly resistance-conferring mutations are rare.

## Background

The genetic complexity of *Plasmodium falciparum *and its ability to generate mutant variants in particular makes it a strikingly successful pathogen. Single nucleotide polymorphisms (SNPs) contribute largely to its genetic variability [1]. *P. falciparum *undergoes mutations in respective target genes, thus resulting in variations of the encoded proteins which facilitate escape from particular antimalarial compounds.

The mitochondrion plays a functional key role in the electron transport system of the parasite [2]. By binding to the *cytochrome bc1 *complex of the parasite, atovaquone, which was introduced in a fixed combination with proguanil hydrochloride as an antimalarial drug in the late 1990s [3], leads to the collapse of the mitochondrial membrane potential at far lower concentrations than that at which the respective mammalian system is affected [4,5]. Its mode of action is unique in targeting parasite mitochondria selectively, thus inhibiting parasitic oxygen consumption [6] without affecting the host's mitochondrial functions [7]. In addition to electron transport inhibition, atovaquone acts through destabilization of the *cytochrome bc1 *complex, thus causing proton leakage to occur through this site. Proguanil enhances this destabilization [8,9].

Atovaquone has been a successful drug against several eukaryotic microbial parasites [10], including *Toxoplasma gondii *[11] and *Pneumocystis carinii *(now *jirovecii*) [12]. However, when used as a single agent against malaria, the drug has shown a high rate of treatment failures [13,14].

Emergence of parasite resistance to atovaquone due to point mutations in the cytochrome b gene has been described [3] and confirmed *in vitro *and *in vivo *[15]. When used as a single agent, slow uptake and high lipophilicity of the drug may result in a prolonged period of parasite exposure to suboptimal atovaquone concentrations [8]. In consequence, atovaquone-resistant parasites appear to emerge frequently due to this suboptimal therapy [13,14,16].

Due to the emergence of resistance against atovaquone as a single therapeutic agent, inclusion of proguanil as a synergistic agent with atovaquone has been developed in order to minimize the occurrence of drug resistance [17]. The combination of atovaquone and proguanil (Malarone^®^) has been found to be effective in treating malaria [13]. While proguanil by itself had no effect on electron transport or mitochondrial membrane potential, it significantly enhanced atovaquone efficacy when used in combination [7,8].

Proguanil, in its prodrug form, acts in synergy with atovaquone by lowering the effective concentration at which atovaquone leads to a collapse of the mitochondrial membrane potential in malaria parasites. The net result is a much lower incidence of treatment failure and resistance emergence, as it has been observed in clinical trials [8]. However, mutant strains of *P. falciparum *were also resistant to the synergistic effects of atovaquone/proguanil combination [7].

Atovaquone/proguanil is in use as an effective, safe and acceptable prophylaxis and oral treatment for uncomplicated malaria in adults and children [18-23]. As it has been already proposed as first-line therapy in Africa [24], the background mutation rate in an area of Eastern and Western Africa (Ethiopia and Gabon) where atovaquone/proguanil has not been widely used outside of treatment studies was determined in this study [25].

## Materials and methods

The clinical material stemmed from clinical studies at both study sites, and ethical approval was obtained from the respective Ethics Committees of Addis Ababa University and of the International Foundation of the Albert Schweitzer Hospital (HAS). Samples from the Ethiopian site were collected at the Jimma Health Center from patients with uncomplicated malaria as described earlier [26]. Forty other samples were collected at the HAS from uncomplicated malaria cases in a study to validate low-dose chemotherapy [27].

### DNA analysis

The DNA extraction procedure was performed using QIA amp^® ^DNA Mini Kit (QIAGEN, Hilden, Germany), according to the manufacturer's instruction. The samples were analysed using nested PCR and DNA sequencing to detect variation in the *cyt b *gene.

To obtain information about mutations in the *cytochrome b *gene we analysed 40 parasite samples from Lambaréné, Gabon. From these samples, the primers Cytb1 (5'-CTCTATTAATTTAGTTAAAGCACAC-3') and Cytb4 (5'-ACAGAATAATCTCTAGCACC-3') were used to amplify a fragment of 939 bp containing the *cyt b *gene. The obtained fragments were analysed on a 1% agarose gel for purity. Gels were stained with CYBR^® ^GREEN I nucleic acid gel stain (Cambrex Bioscience, East Rutherford, NJ, USA) and visualized on a dark reader transilluminator (Clare Chemical Research, Dolores, CO, USA). Prior to sequencing, the amplified DNA was purified by a PCR purification kit (E.Z.N.A.^® ^Cycle – Pure Kit, Erlangen, Germany) following the supplier's instructions. Then, the DNA sequence was determined using Big Dye 1.1^® ^(Applied Biosystems, Foster City, CA, USA) and purified again by DNA grade Sephadex^® ^(Amersham Biosciences AB, Uppsala Sweden). Strand separation was done on an Applied Biosystems Genetic Analyzer 3100 (Foster City, CA, USA). DNA sequences were finally analysed with the Bio-edit sequence alignment program [28] to detect point mutation.

141 samples from Ethiopia were subjected to PCR using the primers CytbF (5'-GGGTATGATACAGCATTAAAAATAC-3') and Cytb4 resulting in a 349 bp fragment. Here we were interested only in the 3' end of the gene since no mutations were detected in the 5' end in the Gabonese samples. The samples were prepared as described above.

## Results and discussion

No mutations were detected in the Ethiopian samples. Single nucleotide polymorphisms (one in each of 4 samples): T676A, C689T; T760G and G925T were detected in the Gabonese samples. Only the latter exchange results also in an amino acid change M to I in one isolate. There were no mutations detected in codon 268.

The antimalarial activity of atovaquone and its enhancer proguanil has been assessed in *in vivo *and *in vitro *drug sensitivity tests, as well as in genetic studies in different countries. In a study from Vietnam, it has been reported that the combination of atovaquone with proguanil (AP) yielded an overall cure rate of 86% to treat recrudescent *P. falciparum *infections that had occurred after primary treatment with other antimalarials. In their study, the authors recommended AP as a safe and promising alternative treatment for *P. falciparum *infections in South-East Asia [29].

In another study, it was described that atovaquone has shown better *in vitro *response as compared to other eight antimalarials among the *P. falciparum *strains collected from 14 countries in South and Central Africa. It was suggested in the same study that atovaquone has a potential to be used as an alternative antimalarial in Africa better than Asian countries [30].

As with other antimalarials, concern about rapid emergence of drug-resistant strains arose following initial reports of definite, or possible, resistance-conferring polymorphisms of the cytochrome b gene found in treatment-failing *P. falciparum*.

Mutations (Tyr268Asn and Tyr268Ser) of the *P. falciparum cytochrome b *gene were reported in cases of malarone treatment failure [15,31]. Sequencing of the cytochrome b-encoding region of mitochondrial DNA together with DNA samples from *P. falciparum *control strains yielded a change from TAT to AAT in codon 268 (Y268N), specifying a change from tyrosine (Tyr) to asparagine (Asn) in an isolate from a Nigerian patient clinically (R1) and *in vitro *resistant to atovaquone/proguanil [31].

A different mutation in this codon (TAT to TCT in codon 268) specifying a change from tyrosine to serine: Y268S was found in a Thai patient with acute atovaquone and pyrimethamine treatment failure [15], and it was also suggested that a change of Tyr268 (Tyr) to Ser268 (Ser) may be a sufficient cause for atovaquone/proguanil treatment failure [32]. Therefore, mutations at codon 268 of the parasite *cytochrome bc1 *gene have been recognized as potential markers to measure and control the emergence of resistance against atovaquone/proguanil treatment [33].

Further single or double amino acid mutations were generated from a cloned line exposed to various atovaquone concentrations in vitro and leading to a significant reduction in parasite susceptibility to atovaquone. These mutations are M133I, K272R, P275T, and G280D [15]. Thus, multiple mutations in the cytochrome b gene are assumed to be more useful in surveillance for atovaquone resistance. Methods that can pick up multiple mutations (such as DNA sequencing) will be appropriate for screening [34]. However, *in vivo *atovaquone resistance may not always be associated with previously identified *cytochrome b *gene point mutations [35] since failure in drug action can also be resulting from metabolic diversion to the alternative respiratory pathway of the parasite [36].

Sequencing of the cytochrome b gene of *P. falciparum *from a patient returning from Mali with Malarone^® ^treatment failure revealed a point mutation at codon 268 (Tyr268Ser). However, there was no detection of alterations at codon 133 of the strains which showed *in vivo *treatment failure even though it has been found, in the same study, in all strains that showed atovaquone resistance *in vitro *[33].

These results underline that in the absence of drug pressure, spontaneous and possibly resistance-conferring mutations are rare. Assessing the activity of atovaquone using *in vitro *drug sensitivity assay and DNA sequencing of the cytochrome *b *gene, Basco and colleagues (2003) did not report any mutations in the target gene in the *P. falciparum *isolates collected from 37 Cameroonians [37]. In addition, using a low nanomolar range of atovaquone, none of the isolates displayed any evidence for atovaquone resistance *in vitro*. In another study, the commonly associated cytochrome b codon 268 mutation has been assessed in 100 isolates of *P. falciparum *from Northern Ghana. None of these isolates exhibited mutation at this position [38]. Pimentel et al. (2006) [39] studied blood samples from 249 atovaquone-proguanil treatment-naïve children and found a prevalence of possibly resistance-conferring polymorphisms in codon 268 in a frequency of < 0.77% (99% significance level).

A low prevalence of Ser268 (0.96%) and Asn268 (0.77%) was also reported in other imported isolates of *P. falciparum *in Europe [32].

Single nucleotide polymorphisms occurred in the Gabonese samples in four of the forty samples (T676A, C689T, T760G, G925T), of which only one led to an amino acid change which has not been associated before with resistance *in vitro *and/or *in vivo*, and which may well not be resistance-conferring. No mutations were detected in the isolates from Ethiopia. This is consistent with those of previous studies from various African countries [37,38]. Musset et al. (2006) [40] genotyped 477 atovaquone-unexposed African *P. falciparum *isolates and found exclusively wild types. Berry et al. (2006) [41] found polymorphisms in 12/135 (8.9%) of unexposed West and Central African isolates; an overall rate which is consistent with ours. Nine were only transitions and therefore 'silent'; only three led to transversions with amino acid changes; again, a rate very to the one presented in this paper.

## Conclusion

In the absence of drug pressure, spontaneous and possibly resistance-conferring mutations are rare. In line with the findings presented here, an elevated rate of resistance against atovaquone is not reported in most of similar studies in endemic areas and in the absence of drug pressure. However, a higher rate of resistance in the future is likely to occur under drug pressure [16,32,36]. As this would be in accordance with the experience with aminoquinoline and sulpha drugs, possibly, probably and definitely resistance-confirming polymorphisms (single, or in combination) will occur with almost certainty.

## Authors' contributions

TG carried out the molecular genetic studies, helped to design the study and contributed to the draft of the manuscript.

AH participated in the design of the study and helped to finalise the manuscript.

PGK participated in the design of the study and helped to finalise the manuscript.

JFK contributed to the molecular genetic studies, helped to design the study and contributed to draft the manuscript.

MPG conceived of the study and contributed to the draft of the manuscript.
